# Deconvolution reveals cell-type-specific transcriptomic changes in the aging mouse brain

**DOI:** 10.1038/s41598-023-44183-7

**Published:** 2023-10-06

**Authors:** Yingxue Ren, Xue Wang, Shuwen Zhang, Hongru Hu, Zachary Quicksall, Sangderk Lee, Josh M. Morganti, Lance A. Johnson, Yan W. Asmann, Na Zhao

**Affiliations:** 1 Department of Quantitative Health Sciences, Mayo Clinic, 4500 San Pablo Road South, Jacksonville, FL 32224 USA; 2 Department of Quantitative Health Sciences, Mayo Clinic, Rochester, MN 55905 USA; 3 Genome Center, University of California, Davis, CA 95616 USA; 4 Sanders Brown Center On Aging, University of Kentucky, Lexington, KY40536 USA; 5 Department of Neuroscience, University of Kentucky, Lexington, KY40536 USA; 6 Department of Physiology, University of Kentucky, Lexington, KY40536 USA; 7 Department of Neuroscience, Mayo Clinic, 4500 San Pablo Road South, Jacksonville, FL 32224 USA

**Keywords:** Computational biology and bioinformatics, Computational neuroscience, Genome informatics, Computational neuroscience

## Abstract

Mounting evidence highlights the crucial role of aging in the pathogenesis of Alzheimer's disease (AD). We have previously explored human apoE-targeted replacement mice across different ages and identified distinct molecular pathways driven by aging. However, the specific contribution of different brain cell types to the gene modules underlying these pathways remained elusive. To bridge this knowledge gap, we employed a computational deconvolution approach to examine cell-type-specific gene expression profiles in major brain cell types, including astrocytes (AS), microglia (MG), oligodendroglia (OG), neurons (NEU), and vascular cells (VC). Our findings revealed that immune module genes were predominantly expressed in MG, OG, and VC. The lipid metabolism module genes were primarily expressed in AS, MG, and OG. The mitochondria module genes showed prominent expression in VC, and the synapse module genes were primarily expressed in NEU and VC. Furthermore, we identified intra- and inter-cell-type interactions among these module genes and validated their aging-associated expression changes using published single cell studies. Our study dissected bulk brain transcriptomics data at the cellular level, providing a closer examination of the cell-type contributions to the molecular pathways driven by aging.

## Introduction

Aging and Alzheimer's disease (AD) involve intricate processes including pathways such as inflammation, lipid dysregulation, mitochondrial dysfunction, and synaptic alterations^1–4^. The progression of these conditions is influenced by multiple cell types, forming a complex network of interactions that underpin disease development. Understanding how these cell types interact is pivotal in deciphering the mechanisms driving AD and other aging-related diseases.

Recent advancements in single-cell and single-nuclei RNA sequencing have significantly enhanced our understanding of diseases at the single cell and cell-type levels^5,6^. However, the high cost associated with these techniques often restricts large-scale experiments necessary for investigating multiple traits of interest with robust statistical power. To address this limitation, deconvolution approaches have emerged, employing computational algorithms to estimate cell-type-specific gene expressions from bulk tissue data, leveraging reference data from single-cell and single-nuclei studies^7–10^. These methods enable the utilization of lower-cost, larger-scale bulk transcriptomics datasets to gain insights into gene interactions across different cell types.

In this study, we applied a cell type deconvolution method to analyze our previously published cerebral cortex transcriptomics data, obtained from male and female apoE-targeted replacement (TR) mice at different ages (3-month-old, 12-month-old, and 24-month-old)^11^. In our previous work, we had reported strong aging-related effects, including immune responses, at bulk transcriptomic level. Using deconvoluted data, our goal of this study was to explore how various brain cell types collectively contribute to critical molecular pathways influenced by aging by examining the cell-type-specific expression of module genes associated with aging. To validate our findings, we conducted comparisons between our deconvoluted data and three single-cell RNA sequencing datasets derived from mice of different ages^12–14^. Our findings highlight the value of computational deconvolution when applied to bulk transcriptomics data, providing novel insights at the cellular level to enhance our understanding of AD and other aging-related diseases.

## Results

### Computational deconvolution of bulk transcriptomics data and identification of major cell types

**Figure 1:**
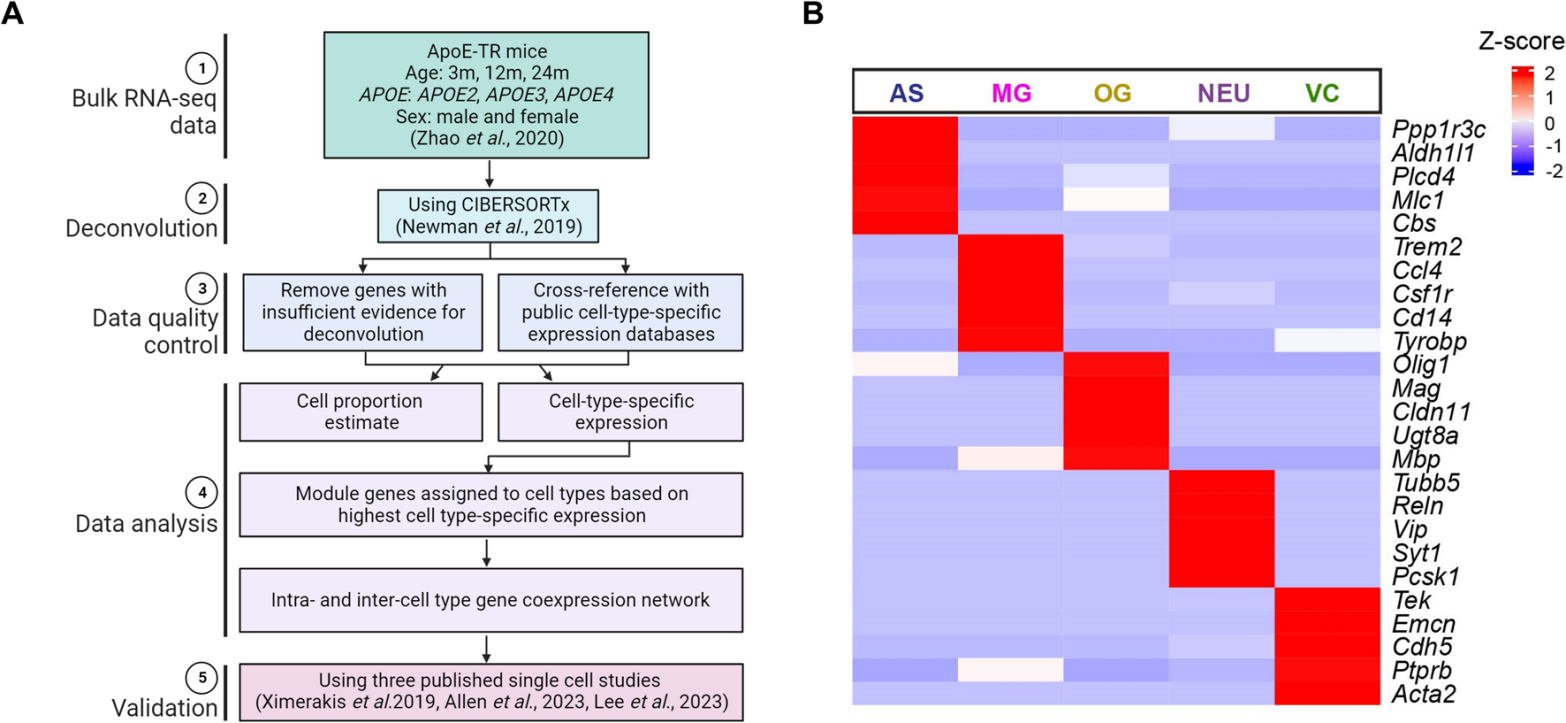
Computational deconvolution of bulk transcriptomics data and identification of major cell types. (**A**) Analytical workflow. (**B**) Heatmap showing the expression of known cell type marker genes deconvoluted from our previously published bulk RNA-seq data.

We employed CIBERSORTx^8^ to perform deconvolution on our previously published bulk transcriptomics data generated from the cerebral cortex of apoE-TR mice^11^ (Fig. 1A, Supplementary Table S1). Our deconvolution process included two primary levels: estimating cell type proportions within each sample and determining transcriptome-wide cell-type-specific expressions within each sample. To ensure the accurate assignment of cell types, we cross-referenced the cell-type-specific expressions of genes with an ensemble of published cell-type-specific data and databases (proteinatlas.org)^15–17^. We then focused on gene modules that showed robust associations with aging. We assigned the genes within these modules to their respective cell types based on their highest cell-type-specific expressions. We then constructed intra- and inter-cell type gene co-expression networks within these modules. This approach allowed us to pinpoint the key cell types involved in the aging-associated gene modules, all while exploring the interactions among genes both within and between cell types. To validate our findings related to aging, we conducted cross-validation using data from three published mouse brain single-cell RNA-seq studies^12–14^.

Our analysis identified five major cell types from the bulk transcriptomics data: astrocytes (AS), microglia (MG), oligodendroglia (OG), neurons (NEU), and vascular cells (VC). To validate the deconvolution results, we compared the expression of cell type markers obtained from our analysis with known cell type marker genes from publicly available data^9^ and confirmed the accuracy of cell type assignments (Fig. 1B).

### Analysis of cell type proportions in relation to aging, *APOE* genotype, and sex

**Figure 2:**
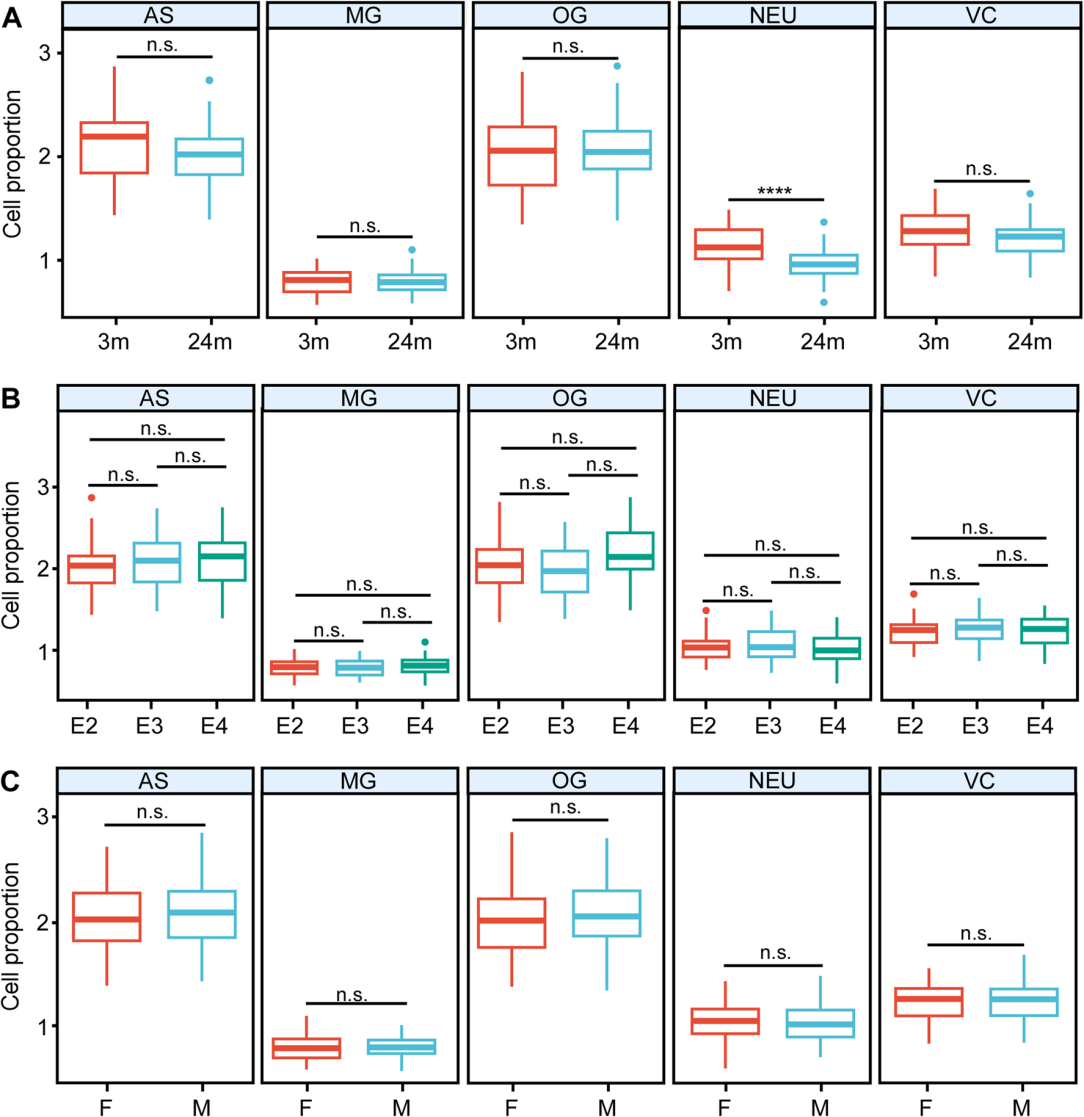
The proportion of the five major brain cell types in each age, *APOE* and sex group. (**A**) The estimated cell type proportions in 3 months (3 m) and 24 months (24 m) mice. (**B**) The estimated cell type proportions in *APOE2* (E2), *APOE3* (E3), and *APOE4* (E4) mice. (**C**) The estimated cell type proportions in female and male mice. T test was used to detect differences between groups. Bonferroni correction was applied to correct for multiple testing. ∗∗∗∗p < 0.0001; *n.s.* not significant.

In our analysis of the deconvoluted data, the initial step was to compare cell type proportions among different age groups, *APOE* genotypes, and between sexes for the five distinct brain cell types. In our prior report^11^, we observed that the transcriptomic differences between 12 and 24 months old were mild, with the most substantial distinction occurring between 3- and 24-month-old brains. Therefore, in this study, our focus was on comparing the two age groups, specifically 3 months and 24 months old. Our analysis revealed that when comparing young and old brains, the only significant difference in cell proportions was within the neuronal population (NEU). Specifically, we identified a significant reduction in the neuronal component at 24 months compared to 3 months, while no significant changes were observed in other cell types in relation to aging (Fig. 2A). This finding is consistent with previous reports demonstrating the loss of neuronal markers, including synaptic proteins, in aged mice compared to young mice^18^. In addition, we did not observe any significant alterations in cell type proportions related to *APOE* genotype (Fig. 2B) or between sexes (Fig. 2C).

### Cell type-specific analysis of gene modules related with immune responses

**Figure 3:**
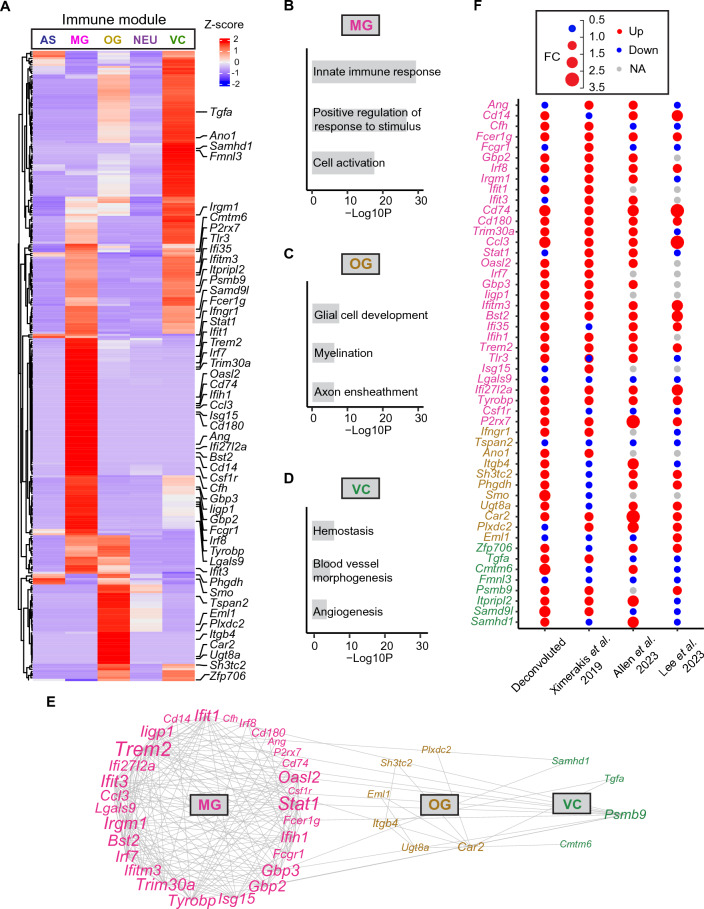
Immune gene module deconvolution. (**A**) Heatmap showing the expression of immune module genes in the five cell types. (**B**) Top GOs enriched in MG genes. All 3 GO terms were based on Biological Processes (BP) (**C**) Top GOs enriched in OG genes based on BP. (**D**) Top GOs enriched in VC genes. Hemostatis was based on Reactome Gene Sets, while Blood vessel morphogenesis and Angiogenesis were based on BP. (**E**) Correlation network of selected module genes from MG, OG and VC. (**F**) Dot plot showing gene fold changes of old versus young animals from our deconvoluted data, Ximerakis et al.^12^, Allen et al.^13^ and Lee et al.^14^. MG genes are denoted in pink, OG genes in gold, and VC genes in green.

The dysregulation of the immune responses is a defining feature of aging^19^ and plays a substantial role in the development of AD^20^. In our prior study, we identified an immune gene module characterized by upregulation with aging and enriched for genes related to immune responses^11^. To gain insights into the specific cell types and their associated genes driving this collective immune response, we assigned the immune module genes to different cell types based on their estimated cell-type-specific expressions (Fig. 3). Our analysis revealed that the immune module genes were predominantly expressed in microglia (MG), oligodendroglia (OG), and vascular cells (VC) (Fig. 3A). Pathway analysis showed that MG genes were enriched for innate immune response (-logP = 29.4), positive regulation of response to stimulus (-logP = 26.7), and cell activation (-logP = 17.6) (Fig. 3B). Among these pathways, well-known MG markers such as *Trem2*, *Tyrobp*, *Cd74*, *Cd14*, and *Csf1r* contributions to these pathways^21^. OG genes also displayed enrichment in immune system processes, sharing genes with MG such as *Stat1*, *Ifit1*, *Ifit3*, and *Appl2*, consistent with the recent findings suggesting that OG is active immunomodulator in response to disease^22,23^. Additionally, OG genes were implicated in specialized pathways including glial cell development (− logP = 7.6), myelination (− logP = 6.2), and axon ensheathment (− logP = 6.1) (Fig. 3C). Key genes in these pathways included *Smo*, *Tspan2*, *Itgb4*, *Ugt8a*, and *Sh3tc2*. Furthermore, numerous VC genes were co-expressed in MG and exhibited functions related to immune responses, with examples including *Gbp2*, *Gbp3*, *P2rx7*, *Trim30a*, and *Fcer1g*. VC-specific genes, on the other hand, displayed enrichment in processes such as hemostasis (-logP = 6), blood vessel morphogenesis (− logP = 4.9), and angiogenesis (− logP = 4) (Fig. 3D). Notable genes in these pathways encompassed *Tgfa*, *Cmtm6*, and *Fmnl3*. The complete pathway analysis results can be found in Supplementary Table S2.

To gain a deeper understanding of the interactions among these genes, we constructed a gene correlation network encompassing the three cell types. Notably, we observed strong connections between MG genes (*Trem2*, *Stat1*, *Tyrobp*, *Trim30a*), OG genes (*Ugt8a*, *Itgb4*, *Car2*), and a VC gene (*Psmb9*) (Fig. 3E).

To validate the age-related expression changes of these key genes and others, we calculated fold changes between old and young animals in MG, OG, and VC, respectively, using our deconvoluted cell-type-specific expressions. We then compared these changes with data from three prominent mouse aging single-cell RNA sequencing studies^12–14^ (Fig. 3F). Specifically, for the study by Ximerakis et al.^12^, we extracted the fold changes between old and young animals in each cell type as listed in their publication. In the studies of Allen et al.^13^, and Lee et al.^14^, we performed differential expression analyses comparing old versus young animals within each cell type using their respective Seurat data objects. Out of the 50 immune genes examined, 31 (62%) displayed the same change in expression direction with age as reported by Ximerakis et al.^12^, 33 (66%) exhibited consistent changes with aging as observed in Allen et al.^13^, and 26 (52%) showed similar age-related changes as reported by Lee et al.^14^. In total, 45 out of these 50 genes (90%) were validated by at least one of the single-cell studies. Additionally, 15 genes (30%) were validated by all three studies. Five genes displayed opposite changes in expression direction with aging compared to all three single-cell studies or were expressed in less than 10% of cells in these studies. These genes include *Ifit3*, *Isg15*, *Csf1r*, *Smo*, and *Plxdc2* (Fig. 3F). It's worth noting that the discrepancies may be attributed to variations arising from factors such as animal sexes, ages, brain regions, tissue preparations, and cell dissociation procedures employed in these single-cell studies.

Collectively, our deconvoluted data indicate that microglia, together with oligodendroglia and vascular cells, play a pivotal role in orchestrating the immune response network within the brain during the aging process.

### Cell type-specific analysis of gene modules related with lipid metabolism

**Figure 4:**
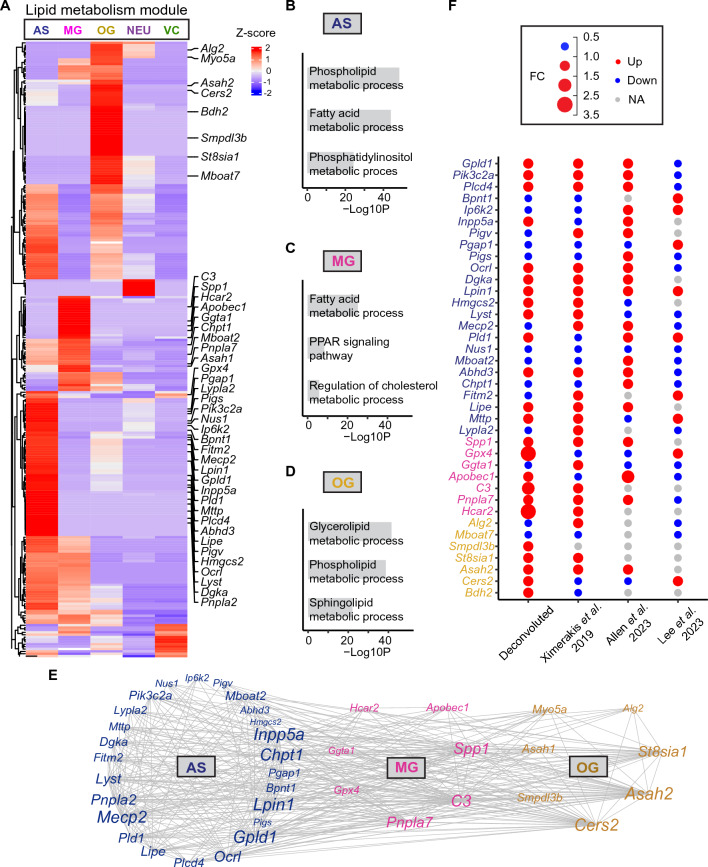
Lipid metabolism gene module deconvolution. (**A**) Heatmap showing the lipid metabolism module genes in the five cell types. (**B**) Top GOs enriched in AS genes. All 3 GO terms were based on Biological Processes (BP). (**C**) Top GOs enriched in MG genes. Fatty acid metabolic process and Regulation of cholesterol metabolic process were based on BP, while PPAR signaling pathway was based on WikiPathways. (**D**) Top GOs enriched in OG genes based on BP. (**E**) Correlation network of selected module genes from AS, MG and OG. (**F**) Dot plot showing gene fold changes of old versus young animals from our deconvoluted data, Ximerakis et al.^12^, Allen et al.^13^, and Lee et al.^14^. AS genes are denoted in dark blue, MG genes in pink, and OG genes in gold.

Lipids play pivotal roles in cell signaling and various physiological processes within the brain and central nervous system. Disruptions in lipid homeostasis and impaired lipid metabolism have been implicated in both aging and AD pathogenesis^3,24^. In our previous study^11^, we identified an upregulated lipid metabolism module (part of the extracellular vesicle module) associated with aging. Given the potential relevance of these genes to aging and AD, we conducted further investigations focusing on this module. Our findings revealed that the lipid metabolism module predominantly comprised genes expressed in astrocytes (AS), microglia (MG), and oligodendroglia (OG) (Fig. 4A). Only a limited number of genes were attributed to neuronal population (NEU) and vascular cells (VC). Specifically, in AS, genes were significantly enriched for processes such as phospholipid metabolism (− logP = 48.4), fatty acid metabolism (− logP = 43.9), and phosphatidylinositol metabolism (− logP = 24.3) (Fig. 4B). Key genes contributing to these pathways included *Mboat2*, *Mecp2*, *Pik3c2a*, *Gpld1*, *Inpp5a*, *Chpt1*, *Ocrl*, and *Lpin1*. In MG, genes were notably enriched for fatty acid metabolism (− logP = 25.1), the PPAR signaling pathway (− logP = 8.4), and the regulation of cholesterol metabolic process (− logP = 5.8) (Fig. 4C). Genes associated with these pathways included *C3*, *Spp1*, *Pnpla7*, *Apobec1*, *Hcar2*, *Ggta1*, and *Gpx4*. OG genes exhibited enrichment in glycerolipid metabolic processes (− logP = 41.5), phospholipid metabolic processes (− logP = 38.7), and sphingolipid metabolic processes (− logP = 22.1) (Fig. 4D). Genes contributing to these pathways encompassed *Cers2*, *Asah1*, *St8sia1*, *Smpdl3b*, and *Alg2*. The complete pathway analysis results can be found in Supplementary Table S2.

Furthermore, our correlation network analysis of module genes across all three major cell types revealed both intra- and inter-cellular interactions. The most robust connections were observed among AS genes (*Inpp5a*, *Chpt1*, *Gpld1*, *Lpin1*, and *Mecp2*), MG genes (*Spp1*, *C3*, and *Pnpla7*), and OG genes (*Asah2* and *Cers2*) (Fig. 4E).

To validate the aging-associated expression changes of these key genes, we again compared fold changes between old and young animals in our deconvoluted data with the three single-cell RNA sequencing studies^12–14^. We found that, out of the 38 lipid metabolism genes, 25 (65.7%) exhibited consistent directional changes with age as reported by Ximerakis et al.^12^, 17 (44.7%) with Allen et al.^13^, and 14 (36.8%) with Lee et al.^14^. In total, 34 out of these 38 genes (89.5%) were validated by at least one single-cell study. Of note, two genes—*Lpin1* and *Nus1*—were validated by all three studies, while four genes displayed opposite aging-related changes compared to these three single-cell studies or were expressed in less than 10% of cells in these single-cell studies, including *Pigv*, *Fitm2*, *Smpdl3b*, and *Bdh2* (Fig. 4F).

Altogether, our deconvoluted data indicate both similarities and differences in lipid dysregulation across various cell types. Further investigation is required to elucidate how these lipid changes influence cellular functions.

### Cell type-specific analysis of gene modules related with mitochondrial function

**Figure 5:**
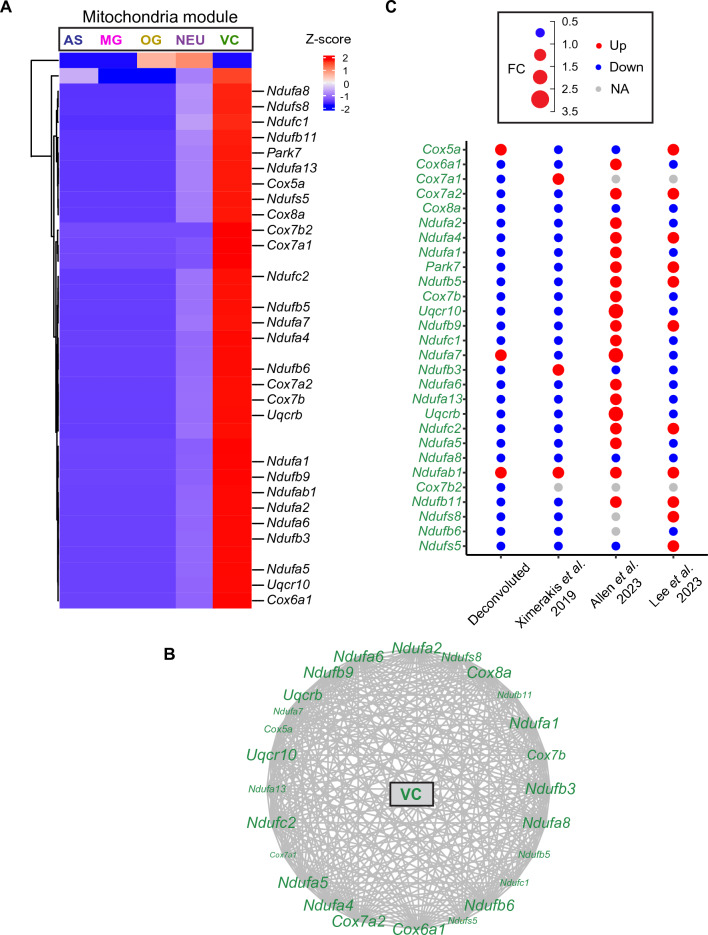
Mitochondria gene module deconvolution. (**A**) Heatmap showing the respiration chain module genes in the five cell types. (**B**) Correlation network of VC genes. (**C**) Dot plot showing gene fold changes of old versus young animals from our deconvoluted data, Ximerakis et al.^12^, Allen et al.^13^, and Lee et al.^14^. VC genes are denoted in green.

Mitochondrial dysfunction and diminished energy metabolism are prominent neuropathological characteristics of AD^25^ and represent key hallmarks of the aging process^26^. In our prior research, we identified a module of genes associated with mitochondria that displayed a downregulation pattern with aging and enriched for respiratory chain pathways. Notably, our deconvolution analyses revealed that these genes were predominantly expressed in vascular cells (VC) (Fig. 5A). This module comprised pivotal hub genes (Fig. 5B), including members of the Cytochrome C Oxidase gene family such as *Cox6a1*, *Cox7a2*, *Cox8a*, and *Cox7b*, alongside genes from the mitochondrial NADH: ubiquinone oxidoreductase gene family, including *Ndufa1*, *Ndufa8*, *Ndufb3*, and *Ndufb6*. Additionally, prominent genes from the Ubiquinol-Cytochrome C Reductase gene family, such as *Uqcrb* and *Uqcr10*, were also featured within this module (Fig. 5B).

The further validation with the three single-cell RNA sequencing studies showed that, among the 28 mitochondria function genes, 23 (82.1%) exhibited concordant changes with age as reported by Ximerakis et al.^12^, 6 genes (21.4%) aligned with Allen et al.^13^, and 16 genes (57.1%) were consistent with Lee et al.^14^ (Fig. 5C). Importantly, 26 out of these 28 genes (92.9%) were validated by at least one single-cell study. Two genes, *Cox7a1* and *Cox7b2*, displayed opposing changes with aging when compared to these three single-cell studies or were expressed in less than 10% of cells in these single-cell studies. It is worth noting that the module of mitochondria-related genes exhibited the most variation when compared to Allen et al.^13^. This divergence is likely due to inadequate sampling of certain vascular cell types, including pericytes and endothelial cells, in the Allen et al.^13^ study, potentially leading to inaccuracies in gene expression profiling.

Overall, the consistent downregulation of these mitochondrial function genes with aging, as reported in our study and corroborated by Ximerakis et al.^12^ and Lee et al.^14^, aligns with the established notion that impaired mitochondrial signaling in the endothelium constitutes an early event in the aging process and contributes causally to the development of various age-related diseases^27^.

### Cell type-specific analysis of gene modules related with synapses

**Figure 6:**
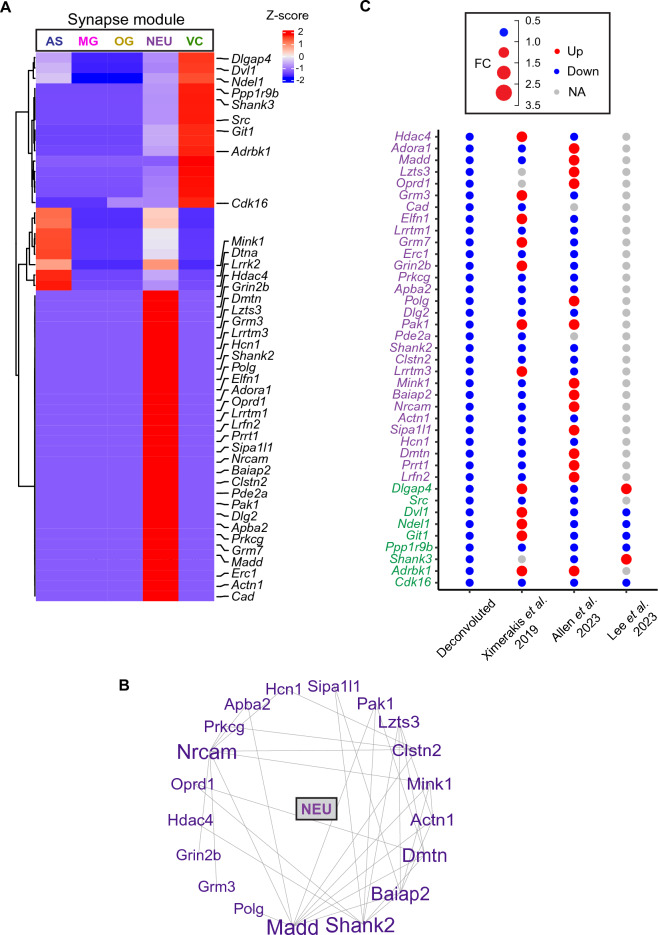
Synapse gene module deconvolution. (**A**) Heatmap showing the synapse module genes in the five cell types. (**B**) Correlation network of NEU genes. (**C**) Dot plot showing gene fold changes of old versus young animals from our deconvoluted data, Ximerakis et al.^12^, Allen et al.^13^ and Lee et al.^14^. NEU genes are denoted in purple, and VC genes in green.

Accumulated research over the years has firmly established that synaptic transmission undergoes changes as individuals age. Indeed, studies have shown that genes regulated by age are associated with synaptic functions^28^. In our investigation, we deconvoluted the synapse module, which exhibited downregulation with aging. Our analysis revealed that the majority of genes within this module were expressed in neurons (NEU), with some also expressed in vascular cells (VC) (Fig. 6A). A subset of genes was associated with astrocytes (AS), demonstrating overlapping expression with the neuronal population (Fig. 6A). Key hub genes within this synapse module included *Madd*, *Shank2*, *Baiap2*, *Nrcam*, and *Dmtn*, all from NEU (Fig. 6B).

In the validation with single-cell RNA sequencing data sets, we found that out of the 30 neuronal genes, 21 (70%) exhibited consistent directional changes with Ximerakis et al.^12^, and 15 (53.3%) with Allen et al.^13^. Of note, there were not many genes captured by Lee et al.^14^ due to the lack of neuronal population in this dataset. In total, 27 out of these 30 neuronal genes (90%) were validated by at least one single-cell study (Fig. 6C). Three genes displayed opposing changes with aging compared to these three single-cell studies or were expressed in less than 10% of cells in these single-cell studies, including *Lzts3*, *Oprd1*, and *Pak1*. Regarding the genes associated with vascular cells (VC), our validation using all three single-cell studies indicated that 3 out of the total 9 (33.3%) VC genes exhibited consistent aging-related changes with Ximerakis et al.^12^, 8 (88.9%) with Allen et al.^13^, and 5 (55.6%) showed similar aging-related changes with Lee et al.^14^. In total, 8 out of 9 VC genes (88.9%) were validated by at least one single-cell study, with gene *Adrbk1* being the only exception, showing opposite changes with aging compared to these three single-cell studies or being expressed in less than 10% of cells in these single-cell studies.

Together, these data provide support for the concept of synaptic alterations occurring with aging, as well as for the critical network changes of neurovascular coupling and neuronal-glial-endothelial interactions observed during aging^29,30^.

## Discussion

In this study, we applied cell type deconvolution to bulk RNA sequencing data from apoE-TR mice to identify key cell types involved in gene modules related to aging. We also explored the molecular pathways and interactions between module genes within and across cell types. Additionally, we identified hub genes with significant connections to others, providing a further understanding of the contributions of different cell types to important aging-regulated pathways. Most of our deconvoluted aging-associated gene expression changes were validated by one or more previously published single cell studies.

During the aging process, the brain undergoes various immune responses, such as neuroinflammation, microglial activation, blood–brain barrier dysfunction, myelin disintegration, immune cell senescence, and altered interactions between immune cells and other brain cells^2^. These immune changes contribute to cognitive decline and increase the risk of neurodegenerative diseases. Our deconvolution data consistently highlights the involvement of microglia, oligodendrocytes, and vascular cells in the immune response during aging. It was reported that microglia and oligodendrocytes have mutual interactions and influence each other's functions^31^. Microglia play a role in modulating immune responses and impacting the survival and differentiation of oligodendrocytes. Additionally, brain vascular cells, including endothelial cells and pericytes, have a dual role in maintaining the integrity of the blood–brain barrier and participating in immune responses^32^. They interact with immune cells and influence inflammation in the brain. Further understanding the intricate crosstalk among these brain cells and their interactions with immune responses is crucial for unraveling disease mechanisms and developing targeted therapies.

Furthermore, our deconvolution analysis reveals dysregulation of lipids in astrocytes, microglia, and oligodendrocytes, with variations in the specific lipid processes involved in each cell type. Both astrocytes and microglia show affected fatty acid metabolism, which has been linked to neuroinflammation^33–35^. It was reported that the pro-inflammatory microglia suppress fatty acid oxidation and synthesis, while anti-inflammatory microglia enhance these processes^35^. Additionally, microglia show dysregulation in the PPAR signaling pathway, known to modulate microglial innate immunity and fatty acid metabolism^36,37^. These findings suggest a potential link between lipid dysregulation and neuroinflammation in astrocytes and microglia. In oligodendrocytes, we observe dysregulation in glycerolipid, phospholipid, and sphingolipid processes. While the understanding of glycerolipid metabolism in oligodendrocytes is limited, it is crucial to acknowledge the significant roles of phospholipids and sphingolipids in myelin composition^38,39^. Importantly, lipid metabolism in oligodendrocytes has been implicated in the development of demyelination, a key factor contributing to the risk of AD^40^. Taken together, these findings highlight age-related lipid dysregulation that may contribute to neuroinflammation and demyelination during the aging process and in AD.

Mitochondrial dysfunction is a key feature of aging, characterized by structural and functional changes in the mitochondria^2^. This leads to reduced energy production, increased production of reactive oxygen species, and impaired mitochondrial quality control. Interestingly, our deconvolution data suggests strong mitochondrial respiration dysregulation in brain vascular cells. It is known that endothelial mitochondrial dysfunction is an important factor causing abnormal function of the endothelium, which plays a central role during atherosclerosis development^41^. These changes can also disrupt the integrity and function of the blood–brain barrier, resulting in increased permeability and inflammation in the brain.

Our deconvoluted data imply that the downregulated synapse module with aging includes genes primarily originating from neurons, vascular cells, and astrocytes. This suggests that during the aging process, it's not just neurons experiencing synaptic loss; the interactions or networks between neurons, glial cells, and vascular cells are also disrupted. This observation aligns well with our current understanding that the brain is a complex tissue, where vascular cells and glial cells closely collaborate with neurons to maintain brain functions^29,30^. These network gene changes can be challenging to detect in single-cell RNA sequencing data if specific cell types are not adequately captured during the cell isolation process.

In our prior study, we reported several gene module changes associated with the *APOE* genotype, including lysosome functions and RNA splicing^11^. However, our deconvolution results indicate that the majority of these genes are attributed to neurons (data not shown). Unfortunately, because the neuronal population was not adequately represented in our single-cell validation dataset^14^, we were unable to validate and present this data in the current study. Therefore, the analysis and validation of *APOE* genotype-related gene module changes will be a subject of future investigation.

It has long been recognized that cell heterogeneity in tissue samples leads to the caveat in bulk RNA sequencing experiments that it is often hard to dissociate true transcriptomics landscape changes from the contribution of cell types. In the study of diseases where certain cell types play a major role in disease pathogenesis, cell-type specific information is particularly pertinent. Computational deconvolution methods have the unique advantage of being readily available as open-source packages, and can be applied to old or previously published data where samples are no longer available or difficult to re-obtain. The success of our application of the deconvolution algorithm in this study serves as proof of principal for the potential of applying computational deconvolution methods to the vast available bulk public dataset to gain novel insights of disease mechanisms.

## Limitations of the study

Our study has several limitations, some of which are inherent to the use of computational deconvolution. First, it is important to acknowledge that computational methods are susceptible to generating false positives. In our case, the transition from bulk gene expression to cell-type-specific gene expression involved multiple layers of machine learning processes, which collectively could lead to inaccurate estimations of gene expression in certain cell types. To mitigate this, it is essential to cross-reference our results with publicly available databases and existing literature, ensuring that we identify and rectify any such estimations and avoid overgeneralization of our findings. For genes with limited public information, experimental validations, such as co-staining, will be crucial to confirm the accuracy of the sample-level deconvolution results in the future. Second, it's important to recognize that different computational deconvolution algorithms may be better suited for specific types of bulk transcriptomics data. Many deconvolution methods have been developed for or evaluated on blood, immune, or tumor samples, and their performance may not be equivalent for tissues with high complexity, such as the brain^7,10,42^. Depending on the specific characteristics of the bulk tissue being studied, it becomes imperative to carefully evaluate the choice of deconvolution method and exercise caution in its application. Acknowledging these limitations, future study should aim to minimize potential errors through rigorous validation strategies. It is also important to critically interpret our findings within the context of existing knowledge, recognizing that computational deconvolution, while a powerful tool, must be employed judiciously and complemented with experimental evidence when necessary.

## Methods

### Dataset description

We used our previously published cerebral cortex transcriptomics data of 141 apoE-TR animals, including male and female *APOE2*, *APOE3* and *APOE4* mice at 3, 12, and 24 months of age^11^. The animal husbandry, tissue processing, RNA extraction, RNA sequencing, quality control and gene count normalization were previously described^11^. The CQN normalized RPKM gene expression values of 19,120 genes were used for deconvolution analyses.

### Cell type deconvolution and gene correlation network analysis

We used the CIBERSORTx algorithm high resolution mode to deconvoluted the bulk transcriptomics data based on the author’s recommendations^8^. Specifically, we used the mouse brain single cell RNA sequencing data from GSE129788 to construct the single cell signature gene matrix due to the data’s close match in both age and brain region to our bulk data^12^. From the cell-type-specific gene expression results, we removed genes that were either “NA” or “− 1”, or had the same deconvoluted expression values across all samples, which indicated insufficient evidence of estimation. Module genes were scaled and visualized via hierarchical clustering in heatmaps using the ComplexHeatmap R package^43^. Pathway analyses of cell type specific module genes were performed using Metascape (https://metascape.org)^44^. Gene correlation network analysis of selected modules were performed using WGCNA^45,46^ and visualized using Cytoscape version v3.10.0^47^.

### Validation of cell-type-specific module genes using public data

To validate the deconvoluted cell-type-specific modules genes, we compared their cell type assignments against the following sources: (1) top 50 mouse brain cell type marker genes from each brain cell type from the BRETIGEA R package, which compared and contrasted five human and mouse cell type-specific transcriptomics datasets to identify consensus brain cell-type marker genes^9^, (2) brain single cell RNA sequencing expression profiles from the Human Brain Atlas v22.0 (proteinatlas.org), (3) published mouse brain MG gene expression profiles across different activation states^15^, (4) published AS gene expression profiles in mouse brain cortical regions^16^, and (5) published OG gene expression profiles across various mouse brain regions^17^.

To validate aging-associated changes in our cell-type-specific module genes, we compared fold changes of old versus young animals between our deconvoluted data and three prominent mouse aging single cell studies^12–14^. For our deconvoluted genes in each cell type, fold changes were calculated between 24 and 3 months using an ANOVA model while adjusting for *APOE* genotype and sex. The fold changes between old (21–23 months) and young (2–3 month) mice from Ximerakis et al.^12^ were obtained from their supplementary Table 6, which was calculated using the MAST model^48^ for each annotated single cell type. To calculate fold changes in Allen et al.^13^, we downloaded the integrated and annotated R data object GSE207848_Cell.rds from CELL x GENE repository (https://cellxgene.cziscience.com/collections/31937775-0602-4e52-a799-b6acdd2bac2e), and used the MAST model to compare 90 weeks (22.5 months) and 4 weeks (1 month) for each single cell type using Seurat version 5^49^. To calculate fold changes in Lee et al.^14^, we obtained the integrated and annotated R data file from the authors and used the MAST model to compare 24 months and 3 months for each single cell type while adjusting for *APOE* genotype using Seurat version 5.

### Supplementary Information

Supplementary Table S1.

Supplementary Table S2.

## Data Availability

The bulk transcriptomics data that were deconvoluted in this study are available on Synapse: https://doi.org/10.7303/syn20808171.
